# Is VA-ECMO a Rescue Technique or a Treatment in Itself for High-Risk Pulmonary Embolism?

**DOI:** 10.31083/j.rcm2307252

**Published:** 2022-07-14

**Authors:** Raphaël Giraud, Benjamin Assouline, Karim Bendjelid

**Affiliations:** ^1^Faculty of Medicine, University of Geneva, 1205 Geneva, Switzerland; ^2^Geneva Hemodynamic Research Group, University of Geneva, 1205 Geneva, Switzerland; ^3^Intensive Care Service, Department of acute medicine, Geneva University Hospitals, 1205 Geneva, Switzerland

**Keywords:** ECMO, high-risk pulmonary embolism, cardiogenic shock

High-risk pulmonary embolism (PE) is defined by haemodynamic instability, which can manifest as cardiac arrest (CA), associated with either CT-scan confirmation of PE and/or echocardiographic signs of right ventricular (RV) dysfunction [1]. Risk stratification is essential to determine the optimal therapeutic management [1]. In the acute phase of high-risk PE, treatment should combine haemodynamic and respiratory support associated with anticoagulation and a reperfusion strategy [1]. In the most critical cases, initial resuscitation now includes the use of mechanical circulatory support techniques, mostly venoarterial extracorporeal membrane oxygenation (VA-ECMO). Even if there is currently no randomized controlled study testing its efficacy and safety in the context of high-risk PE, this technique is widely used, with encouraging results [2], even as stand-alone therapy [3]. Indeed, ECMO allows, on the one hand, the unloading of the failing right ventricle and, on the other hand, both perfusion and oxygenation of the organs [3]. This technique is invasive and has its own complications, in particular haemorrhage, especially following systemic thrombolysis (ST) [4].

In Issue 6 of this journal, Ltaief Z *et al*. [5] published a retrospective study of 18 patients implanted with VA-ECMO for high-risk PE. In their cohort, only two patients did not experience cardiac arrest (CA) (11%). Five were implanted with ECMO after return of spontaneous circulation (ROSC) and 11 during cardio-pulmonary resuscitation (CPR). Concerning reperfusion treatments, eight patients (44%) received only anticoagulation, 9 (50%) systemic thrombolysis (ST), 4 surgical embolectomy (SE) and only one patient catheter-directed thrombolysis (CDT). ICU survival was 22%, 9% in the ECPR group and 42% for patients implanted in refractory shock. The main causes of death were postanoxic encephalopathy (39%), intractable shock and intractable bleeding (17% each). In the end, all the cannulated patients who presented CA died, and the hospital survival in the other patients was 42%, bringing the total survival of this cohort to 17%. Although the no-flow time was relatively short (<2 min) in all patients, the low-flow time was significantly longer in nonsurvivors. The authors also observed that bleeding complications were more frequent in cases of systemic thrombolysis.

These results highlight that high-risk PE remains a potentially fatal disease and a therapeutic challenge, even in a tertiary centre with a full curative arsenal. They confirm that CA brings an increased risk of death. Indeed, several meta-analyses and retrospective studies corroborate these results [2, 4]. The poor neurological outcome following CPR in the context of PE can be explained as follows: the obstruction of the pulmonary arteries induces, on the one hand, the decrease in the effectiveness of cardiac massage, reducing the transpulmonary flow, the preload left ventricular and therefore cardiac output and cerebral perfusion, and, on the other hand, an increase in central venous pressure that induces a decrease in cerebral blood return [6], an increase in cerebral blood volume and therefore a higher intracranial pressure [7]. In parallel, PE induces a disorder of haematosis that also aggravates cerebral hypoxia [7]. Therefore, it appears essential that CPR be started immediately and that the implantation of ECMO be as rapid as possible, shortening the low-flow time to its minimum [8].

High-risk PE, as well as VA-ECMO, requires systemic anticoagulation. In addition, international guidelines recommend the use of reperfusion therapy [1]. Systemic thrombolysis, which may allow a rapid reduction in vascular obstruction and therefore an improvement in RV failure, would be the first choice in unstable patients or even in CA before the establishment of VA-ECMO. However, ST is highly associated with the risk of bleeding (20% risk of major bleeding, 2% risk of intracranial bleeding) [9], and it is sometimes contraindicated [10] and sometimes ineffective (8% failure) [11]. Obviously, it no longer has a place when ECMO is implanted.

Catheter-directed therapy appears to be a promising technique for allowing clot reduction and haemodynamic improvement with smaller doses of fibrinolytics than ST and therefore a lower risk of bleeding. On the other hand, few studies on it are available [1], CDT requires expertise in endovascular techniques, and haemodynamic improvement can only be expected after several hours of treatment [10]. Therefore, this technique does not seem to be suitable for the most unstable patients, a fortiori in CA, but CDT may have a place if combined with VA-ECMO by allowing accelerated recovery of RV function with minimal complications [12].

Surgical embolectomy (SE) allows, by the incision of the two pulmonary arteries, the ablation or aspiration of fresh clots. SE is one of the therapeutic options in high-risk PE, especially for patients with absolute contraindications to ST or in cases of failure of ST or CDT, and complications occur in 7% of SEs [13]. Even if this technique appears to be feasible, safe and effective for RV recovery [14], SE must be combined with ECMO in patients who are too unstable or patients in CA who would not tolerate this surgery without haemodynamic support [3]. Moreover, it is only possible in an experienced heart surgery centre [15]. Finally, the absence of a dedicated randomized study means that the level of evidence for its use remains low [10].

Therefore, we congratulate Ltaief Z *et al*. [5] for the interesting clinical investigation. An essential question remains: Should a reperfusion technique, which has its own complications, be done in patients already implanted with VA-ECMO? Further dedicated studies are necessary to answer this fundamental issue.

## References

[1] Konstantinides SV, Meyer G, Becattini C, Bueno H, Geersing G, Harjola V (2020). 2019 ESC Guidelines for the diagnosis and management of acute pulmonary embolism developed in collaboration with the European Respiratory Society (ERS). *European Heart Journal*.

[2] Scott JH, Gordon M, Vender R, Pettigrew S, Desai P, Marchetti N (2021). Venoarterial Extracorporeal Membrane Oxygenation in Massive Pulmonary Embolism-Related Cardiac Arrest: a Systematic Review. *Critical Care Medicine*.

[3] Giraud R, Banfi C, Siegenthaler N, Bendjelid K (2016). Massive pulmonary embolism leading to cardiac arrest: one pathology, two different ECMO modes to assist patients. *Journal of Clinical Monitoring and Computing*.

[4] Giraud R, Laurencet M, Assouline B, De Charrière A, Banfi C, Bendjelid K (2021). Can VA-ECMO Be Used as an Adequate Treatment in Massive Pulmonary Embolism. *Journal of Clinical Medicine*.

[5] Ltaief Z, Lupieri E, Bonnemain J, Ben-Hamouda N, Rancati V, Schmidt Kobbe S (2022). Venoarterial Extracorporeal Membrane Oxygenation in High-Risk Pulmonary Embolism: a Case Series and Literature Review. *Reviews in Cardiovascular Medicine*.

[6] Bendjelid K (2005). Right Atrial Pressure: determinant or result of change in venous return. *Chest*.

[7] Banfi C, Assouline B, Bendjelid K, Giraud R (2022). Can a Heart Recently Recovered from an Acute Pulmonary Embolism Supported by Venoarterial Extracorporeal Membrane Oxygenation be Considered for Donation. *ASAIO Journal*.

[8] De Charrière A, Assouline B, Scheen M, Mentha N, Banfi C, Bendjelid K (2021). ECMO in Cardiac Arrest: A Narrative Review of the Literature. *Journal of Clinical Medicine*.

[9] Abraham P, Arroyo DA, Giraud R, Bounameaux H, Bendjelid K (2018). Understanding haemorrhagic risk following thrombolytic therapy in patients with intermediate-risk and high-risk pulmonary embolism: a hypothesis paper. *Open Heart*.

[10] Delmas C, Aissaoui N, Meneveau N, Bouvaist H, Rousseau H, Puymirat E (2020). Reperfusion therapies in pulmonary embolism-state of the art and expert opinion: A position paper from the ”Unite de Soins Intensifs de Cardiologie” group of the French Society of Cardiology. *Archives of Cardiovascular Diseases*.

[11] Meneveau N, Seronde M, Blonde M, Legalery P, Didier-Petit K, Briand F (2006). Management of Unsuccessful Thrombolysis in Acute Massive Pulmonary Embolism. *Chest*.

[12] Tran D, Hays N, Shah A, Pasrija C, Cires‐Drouet RS, Toursavadkohi SA (2021). Ultrasound‐assisted catheter directed thrombolysis for pulmonary embolus during extracorporeal membrane oxygenation. *Journal of Cardiac Surgery*.

[13] Kalra R, Bajaj NS, Arora P, Arora G, Crosland WA, McGiffin DC (2017). Surgical Embolectomy for Acute Pulmonary Embolism: Systematic Review and Comprehensive Meta-Analyses. *the Annals of Thoracic Surgery*.

[14] Goldberg JB, Spevack DM, Ahsan S, Rochlani Y, Dutta T, Ohira S (2020). Survival and Right Ventricular Function after Surgical Management of Acute Pulmonary Embolism. *Journal of the American College of Cardiology*.

[15] Hartman AR, Manetta F, Lessen R, Pekmezaris R, Kozikowski A, Jahn L (2015). Acute surgical pulmonary embolectomy: a 9-year retrospective analysis. *Texas Heart Institute Journal*.

